# An unusually subtle presentation of chromoblastomycosis

**DOI:** 10.1016/j.jdcr.2023.07.029

**Published:** 2023-08-03

**Authors:** William C. Lau, Yasin Damji, Gregory M. Orlowski

**Affiliations:** a Department of Dermatology, Boston Medical Center, Boston, Massachusetts; b Boston University Chobanian & Avedisian School of Medicine, Boston, Massachusetts

**Keywords:** chromoblastomycosis, deep fungal infection, fungus

## Introduction

Chromoblastomycosis (CBM) is an uncommon implantation mycosis caused by pigmented fungi, most commonly *Fonsecaea* spp. and *Cladophialophora* spp.^1^ The World Health Organization classifies CBM as a neglected tropical and occupational disease as it primarily affects the lower extremities of middle-aged men in low and middle-income countries, where the fungi is acquired through cutaneous injuries.^2^, ^3^, ^4^, ^5^ CBM is often found in rural, tropical, or subtropical environments, and it is the most prevalent in Latin America, Asia, Africa, and the Caribbean.^1^^,^^3^^,^^5^ In this paper, we report a case of CBM with a particularly subtle clinical presentation.

## Case report

A 52-year-old man with diabetes presented with a lesion on the tip of his right index finger. The lesion was present for 8 years. It grew to the current size several years ago but had since remained unchanged. He denied previous trauma or associated systemic symptoms. However, the lesion was somewhat tender, prompting a referral to dermatology. The patient lived in the Northeastern United States for decades after immigrating from the Dominican Republic (DR) at the age of 13 years. He traveled back to the DR biannually, visiting beaches and lakes. He also reported a distant history of frequent gardening work with his father in the DR.

**Fig 1:**
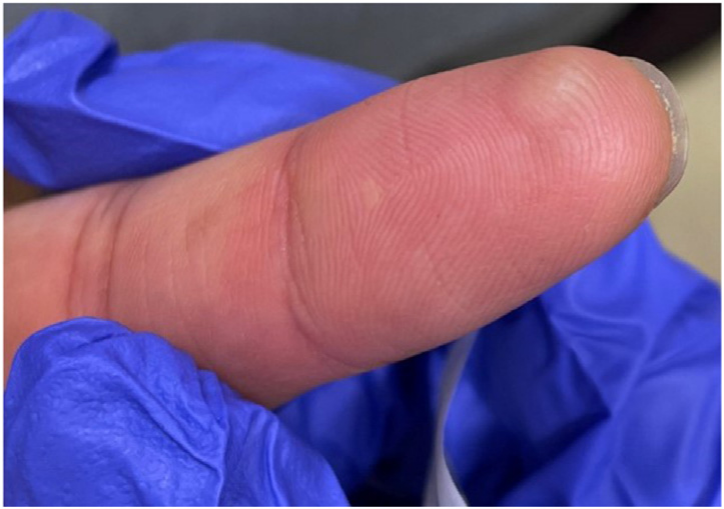
Nonerythematous, nonscaly, smooth, pale-to-skin-colored, dome-shaped papule measuring 5 mm on the right index finger.

On physical examination, the ventral surface of his right index finger had a 5 mm, well-demarcated, firm, smooth, nonscaly, nonerythematous, slightly tender, pale-to-skin-colored, dome-shaped papule (Fig 1).

**Fig 2:**
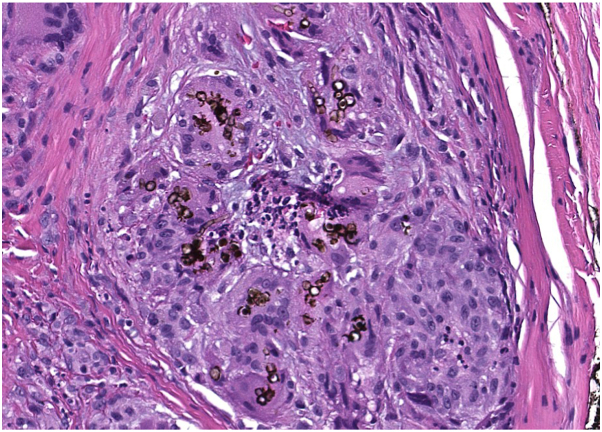
Microscopic examination of the biopsy site (stained with hematoxylin-eosin plus Grocott methenamine silver) revealed pigmented sclerotic bodies (*Medlar bodies*) and septate fungal hyphae at high magnification.

The differential diagnosis included a callus, glomangioma or glomus, mucoid cyst, and acral digital fibrokeratoma. A 4-mm punch biopsy was performed, revealing a nodular dermal aggregate of epithelioid histocytes and multinucleated giant cells forming epithelioid granulomas containing brown-staining thick-walled structures characteristic of sclerotic bodies and septate fungal hyphae, consistent with CBM (Fig 2). Given the low suspicion for a deep fungal infection, a tissue culture was not obtained at the time of biopsy. The lesion was considered to be cleared following biopsy, and no further treatment was recommended after a reassuring evaluation by infectious disease. The patient was instructed to monitor his finger and return to the clinic if the lesion recurred.

## Discussion

The presentation of CBM can be diagnostically challenging, as illustrated by the subtle lesion described in this report. Classically, CBM presents acutely, most often on the lower extremities, as an erythematous macule that gradually develops into a pink papule over time.^5^^,^^6^ Inoculation is most commonly observed on the lower extremities, differing from the present case. Occasionally, these lesions can heal as sclerotic plaques, scars, or keloids.^5^ Scratching can result in the spread of these lesions to other body locations via autoinoculation. Chronic lesions can develop into nodular, verrucous, tumorous, cicatricial, and plaque-like growths and may even mimic other diseases including but not limited to ecthyma, eumycetomas, lacaziosis, and verrucae.^5^^,^^6^

A missed diagnosis can lead to many CBM complications such as bacterial infections, lymphedema, ankylosis, and carcinogenesis.^4^ However, reaching the required level of suspicion for diagnosis is challenging. Even with more classic presentations, the time required for diagnosis can range from 1 month to nearly 40 years after the initial infection.^4^ As a result, the true incubation time of CBM is unknown and may vary depending on the fungal pathology, further complicating estimates of inoculation time.^7^ Moreover, the literature regarding the clinical characteristics of CBM in specific areas, such as the DR, is extremely limited, warranting further research into characterizing the true disease burden and infectious epidemiology, particularly within the low and middle-income countries.^3^ Therefore, given these limitations, reaching a diagnosis with such a subtle presentation as described for our patient, before the spread of any satellite lesions beyond the primary lesion on his finger, requires enough clinical suspicion to prompt a biopsy. Ideally, a tissue culture should also be included to allow for species identification. Here we can only speculate that this patient was most likely infected by *Fonsecaea* spp., given the humid, tropical climate of the DR and the overwhelming majority of CBM cases caused by *Fonsecaea* spp. (90%).^4^ In this case, the histopathology and clinical correlation were sufficient for this critical diagnosis and differentiating CBM from other diseases.^6^

Here, the patient’s small, localized lesion was cleared during biopsy. If not cleared by biopsy, excision is recommended for small, well-demarcated primary lesions.^6^ Had the disease progressed beyond the primary site, the treatment for CBM could have included surgical excision, cryotherapy, laser surgery, and/or systemic antifungal treatment with itraconazole or terbinafine.^6^ However, chronic and extensive CBM may become refractory to initial treatments, requiring long-term systemic antifungal therapy, and cure can be difficult to confirm.^6^

In summary, the present case depicts a seldom-reported subtle manifestation of CBM that could have been easily overlooked. In contrast to the prominent, pigmented, and verrucous lesions on the lower extremities more often described in the literature, this patient’s biopsy-proven CBM on the finger was a relatively asymptomatic (only mildly tender), solitary, nonpigmented, and smooth papule, resembling a callus. To avoid delays in patient care that may lead to progression and further morbidity, clinicians should be suspicious of seemingly innocuous early presentations of CBM in patients who frequently visit or are originally from endemic areas with an unexplained, persistent acral papule.

## Conflicts of interest

None disclosed.
